# Draft Genome Sequences of 53 Genetically Distinct Isolates of *Bordetella bronchiseptica* Representing 11 Terrestrial and Aquatic Hosts

**DOI:** 10.1128/genomeA.00152-15

**Published:** 2015-04-23

**Authors:** Karen B. Register, Yury V. Ivanov, Nathan Jacobs, Jessica A. Meyer, Laura L. Goodfield, Sarah J. Muse, William E. Smallridge, Lauren Brinkac, Maria Kim, Ravi Sanka, Eric T. Harvill, Liliana Losada

**Affiliations:** a National Animal Disease Center, USDA, Agricultural Research Service, Ames, Iowa, USA; b The Department of Veterinary and Biomedical Sciences, Pennsylvania State University, University Park, Pennsylvania, USA; c J. Craig Venter Institute, Rockville, Maryland, USA

## Abstract

*Bordetella bronchiseptica* infects a variety of mammalian and avian hosts. Here, we report the genome sequences of 53 genetically distinct isolates acquired from a broad range of terrestrial and aquatic animals. These data will greatly facilitate ongoing efforts to better understand the evolution, host adaptation, and virulence mechanisms of *B. bronchiseptica*.

## GENOME ANNOUNCEMENT

*Bordetella bronchiseptica* infects a variety of terrestrial and aquatic animals, having a host range that is remarkably broad compared with that of other *Bordetella* species. Most frequently, it causes respiratory disease in pigs and dogs, but is also an occasional zoonotic pathogen (1, 2). For some hosts, including birds and several species of wild mammals, its colonization has not been associated with disease (3, 4). The characterization of isolates using multilocus enzyme electrophoresis (5), PvuII ribotyping (6–8), and multilocus sequence typing (8, 9) indicates that some genotypes preferentially infect one or a few hosts. Here, we report the genome sequences of 53 *B. bronchiseptica* isolates selected to maximally represent geographic, host range, and molecular diversity. The isolates originated from terrestrial and aquatic hosts including 10 mammalian and one avian species, and they collectively represent Australia, Asia, Europe, and North America. They include the 32 PvuII ribotypes so far defined (6–8) (K. B. Register, unpublished data), 29 of the 60 multilocus sequence types (STs) currently identified among the *B. bronchiseptica* species (9) (http://pubmlst.org/bordetella/), and 5 STs not previously associated with the bacterium.

Genomic DNA was prepared (10) and sequenced using a combination of 3- or 5-kb mate-pair Illumina MiSeq 2 × 250-bp and HiSeq 2000 1 × 100-bp paired-end reads. After quality trimming, the reads for each strain (between 2,221,299 and 6,288,700) were assembled with the Celera Assembler 6.1 (11) or the Velvet assembler (12). The underlying consensus sequences and gaps were improved using custom scripts to recruit unmapped reads. All the genomes have between 41 and 296 contigs (median, 117 contigs) (Table 1), with *N*_50_ values ranging from 41,868 bp to 281,818 bp (median, 100,254 bp). The overall G+C content is ~68.1%, with genome sizes ranging from 5.04 Mb to 5.83 Mb. The genomes were annotated using the J. Craig Venter Institute (JCVI) prokaryotic annotation pipeline and contain between 4,388 and 5,660 predicted protein-coding genes. Due to their high copy number, the rRNA loci were broken in the assemblies, so the exact number of operons could not be confidently enumerated in each strain. All strains have between 50 and 68 tRNAs, consistent with previously published genomes (13). The pangenome of the species was estimated at 10,375 genes, with just over 3,300 genes present in all strains, and an additional 1,084 genes present in >90% of the strains. In contrast, just over 3,300 singleton genes were identified, with a single strain containing as few as 1 and as many as 310 strain-specific genes. The majority of the unique genes encode hypothetical proteins or proteins with functions associated with phage and other mobile elements. These results are typical of species that frequently acquire genes by horizontal transfer.

Whole-genome single-nucleotide polymorphism (SNP) analysis clusters isolates into two main groups corresponding to complexes I and IV (9). All avian isolates cluster in complex IV, while most other nonhuman isolates cluster in complex I. In contrast, the human isolates from North America and Europe are evenly dispersed between complexes I and IV. The results of this study provide a wealth of information useful for understanding the evolution, host adaptation, and virulence mechanisms of *B. bronchiseptica*.

### Nucleotide sequence accession numbers.

The sequences of the *B. bronchiseptica* isolates have been deposited in GenBank under the accession numbers listed in Table 1; the source of each strain is also listed.

**TABLE 1:** Strain descriptions and genome assembly characteristics

*B. bronchiseptica* strain	Host	No. of contigs	Length (bp)	GenBank accession no.	Repository
00-P-2730	Human	296	5,831,418	JGWG00000000	NRRL
00-P-2796	Human	179	5,551,792	JGWH00000000	NRRL
345	Human	79	5,286,504	JGWJ00000000	Harvill Lab
3E44	Rabbit	104	5,255,953	JGWK00000000	NRRL
7E71	Horse	109	5,163,549	JGWL00000000	NRRL
980	Unknown	70	5,535,898	JGWM00000000	Harvill Lab
A1-7	Rabbit	184	5,265,200	JGWO00000000	Harvill Lab
B18-5	Rabbit	99	5,219,442	JGWP00000000	Harvill Lab
B20-10725633	Rabbit	124	5,316,022	JGWQ00000000	Harvill Lab
CA90 BB02	Turkey	170	5,126,467	JHBU00000000	Harvill Lab
CA90 BB1334	Turkey	120	5,269,162	JGWR00000000	NRRL
CARE970018BB	Pig	121	5,240,190	JGWS00000000	NRRL
D756	Human	102	5,233,656	JGWT00000000	Harvill Lab
D989	Human	75	5,326,241	JGWU00000000	Harvill Lab
D993	Human	197	5,278,699	JGWV00000000	Harvill Lab
E010	Human	187	5,179,093	JGWW00000000	Harvill Lab
E012	Human	156	5,175,924	JGWX00000000	Harvill Lab
E013	Human	119	5,099,096	JGWY00000000	Harvill Lab
E014	Human	111	5,210,402	JGWZ00000000	Harvill Lab
F-1	Turkey	96	5,377,336	JGXA00000000	NRRL
F2	Turkey	164	5,380,523	JGXB00000000	NRRL
F4563	Human	162	5,263,073	JGXC00000000	NRRL
GA96-01	Human	158	5,292,152	JGXD00000000	NRRL
M435/02/3	Seal	182	5,157,766	JGXE00000000	NRRL
M85/00/2	Seal	159	5,157,897	JGXF00000000	NRRL
MBORD591	Dog	271	5,151,134	JGXG00000000	NRRL
MBORD595	Dog	107	5,214,983	JGXH00000000	NRRL
MBORD624	Horse	213	5,306,540	JGXI00000000	NRRL
MBORD632	Horse	164	5,148,641	JGXJ00000000	NRRL
MBORD635	Cat	55	5,092,496	JGXK00000000	NRRL
MBORD665	Guinea pig	51	5,147,090	JGXL00000000	NRRL
MBORD668	Guinea pig	59	5,149,790	JGXM00000000	NRRL
MBORD670	Guinea pig	80	5,169,029	JGXN00000000	NRRL
MBORD675	Human	41	5,173,023	JGXO00000000	NRRL
MBORD678	Guinea pig	50	5,184,788	JHBQ00000000	NRRL
MBORD681	Koala	70	5,158,310	JGXP00000000	NRRL
MBORD698	Koala	52	5,151,735	JGXQ00000000	NRRL
MBORD707	Turkey	56	5,138,088	JGXR00000000	NRRL
MBORD731	Horse	52	5,132,633	JGXS00000000	NRRL
MBORD762	Guinea pig	63	5,280,420	JHBR00000000	NRRL
MBORD782	Cat	90	5,134,438	JGXT00000000	NRRL
MBORD785	Dog	102	5,152,034	JGXU00000000	NRRL
MBORD839	Dog	103	5,190,832	JGXV00000000	NRRL
MBORD849	Pig	68	5,216,172	JGXW00000000	NRRL
MBORD901	Turkey	109	5,096,955	JGXX00000000	NRRL
MO211	Human	140	5,258,097	JHOJ00000000	Harvill Lab
MO275	Human	131	5,032,460	JHBS00000000	NRRL
OSU054	Turkey	131	5,400,773	JHBZ00000000	Harvill Lab
OSU095	Turkey	54	5,460,040	JGXY00000000	NRRL
OSU553	Turkey	250	5,685,971	JGXZ00000000	NRRL
RB630	Rabbit	48	5,312,681	JGYA00000000	NRRL
SBL-F6116	Human	121	5,060,248	JHBT00000000	NRRL
SO10328	Sea otter	115	5,116,835	JGYB00000000	NRRL
